# Dietary patterns of Filipino older adults and associated factors: analysis of the 2013 National nutrition survey and 2018–2019 expanded National nutrition survey

**DOI:** 10.1186/s12877-025-06426-2

**Published:** 2025-10-23

**Authors:** Robby Carlo Tan, Kyler Kenn Castilla, Michael Serafico, Marco Mensink, Lisette CPGM de Groot

**Affiliations:** 1 Department of Science and Technology, Food and Nutrition Research Institute, Taguig City, Metro Manila Philippines; 2 Division of Human Nutrition & Health, Wageningen University & Research, Wageningen, the Netherlands

**Keywords:** Ageing, Dietary pattern, Nutrition, Older adults, Philippines

## Abstract

**Background:**

The Philippines is transitioning into an ageing society soon. Nutrition is one of the key determinants of healthy ageing and understanding the dietary pattern of older adults is an important step for public health practice. This study describes the dietary patterns of older Filipino adults and determines the associated factors using the 2013 National Nutrition Survey (NNS) and 2018–2019 Expanded NNS (ENNS) datasets.

**Methods:**

From both surveys, a combined total of 11,306 older adults (≥ 60 years old) were included in the analyses. Food items from the two non-consecutive 24-h food recalls were classified into 17 food groups and were analyzed using principal component analysis (PCA). Factor scores derived from the PCA were used to determine adherence to the generated dietary patterns. Multivariate binary logistic regression was used to determine factors associated with adherence to the dietary patterns.

**Results:**

Three major dietary patterns, similar for both NNS and ENNS, were identified namely (1) meat-based; (2) traditional rice and fish; and (3) vegetables, fruits and oil pattern. For both surveys, the odds of adherence to the meat-based pattern were significantly lower among females, adults aged 70 years and older, rural dwellers, and those with low BMI, lower educational attainment, and lower socioeconomic status. Sociodemographic, nutritional, and lifestyle factors were also associated in varying degrees with adherence to the traditional and vegetables, fruits, and oil patterns.

**Conclusion:**

Our study showed that the dietary patterns of older adults in the Philippines were associated with sociodemographic factors, lifestyle, and nutritional status. This study sheds light on understanding better the role of sex, age, SES, educational attainment, and lifestyle factors, nutritional status in influencing dietary patterns of older adults. Our findings make a valuable contribution in crafting specific and timely nutrition-focused interventions for the older population in support towards healthy aging.

**Supplementary Information:**

The online version contains supplementary material available at 10.1186/s12877-025-06426-2.

## Introduction

The global population of people aged 65 years and older is expected to increase from 10 to 16% by 2050 [1]. In Europe and Northern America, the number of older adults is projected to grow from 200.4 million in 2019 to 296.2 million in 2050. However, East and Southeast Asia will see the largest increase in older adult population from 260.6 million in 2019 to 572.5 million in 2050 [2].The Philippine Statistics Authority (PSA) reported that older adults aged 60 years and above comprised 8.5% (9.22 million) of the Household population in 2020 [3], and is projected to reach 12.8% by 2035 [4]. This puts a spotlight on the health needs of the older Filipino population who are faced with an increased risk of developing age and lifestyle-related diseases such as cardiovascular [5, 6], neurocognitive [7–9] and musculoskeletal diseases [10].

Results from the Philippines National Nutrition Survey (NNS) reported that one in every three (35%) older adults had elevated blood pressure and one in every seven (13.8%) had high fasting blood glucose level [11]. Impacts on health accompanied by ageing are usually chronic and complex which is why improving health and quality of life should be a primary goal amidst this ageing population [12].

Diet plays a crucial role in the quality of life of older adults [13]. Physiological, psychosocial and economic factors that accompany old age may influence food choices of older adults that can lead to poorer diet quality and eventually, impaired nutritional status [14, 15]. Investigating dietary changes in the older population caused by these factors may possibly explain the relationship between dietary patterns and certain health outcomes.

Studies on Filipino dietary patterns have been conducted recently focusing on adults aged 20 years and older [16–18]. However, no local studies have assessed the dietary patterns specifically for the older population. It is of prime importance in the Philippines to understand the diet of older adults to contribute to crafting and improving targeted policies and programs for the ageing Filipino population. Hence, this study was undertaken to describe the dietary patterns of older Filipino adults and explore associated factors using the 2013 NNS and 2018–2019 Expanded National Nutrition Survey (ENNS) datasets. A posteriori approach using principal component analysis (PCA), a method used in determining population-specific dietary patterns [19], was used to analyze the dietary data to identify possible associations between dietary patterns and certain health outcomes [20].

## Methods

### Study design and population

This study analyzed the 2013 NNS and 2018–2019 ENNS dataset of the Department of Science and Technology - Food and Nutrition Research (DOST-FNRI). The NNS, a cross-sectional survey, is one of the prime and most comprehensive surveys since the first Nutrition Survey in 1978, generating primary data on food and nutrition in the Philippines. The 2013 NNS adopted the 2003 master sample of the PSA which employed a stratified three-stage sampling design covering the 17 regions and 80 provinces of the country [21]. Meanwhile, the ENNS utilized the 2013 Master Sample of the PSA which is a two-stage cluster sampling design with enumeration areas (EA) or group of adjacent small EAs as the primary sampling units, followed by the selection of secondary sampling units composed of housing units/households and was conducted from the year 2018 until March 2020 due to the COVID-19 pandemic restrictions [11]. The 2013 NNS and 2018–2019 ENNS were independent nationally representative cross-sectional surveys utilizing different sampling frames [22]. Both surveys were approved by the FNRI Institutional Ethics Review Committee. Details of both survey sampling design and methodology have been previously published [11, 21].

**Fig. 1:**
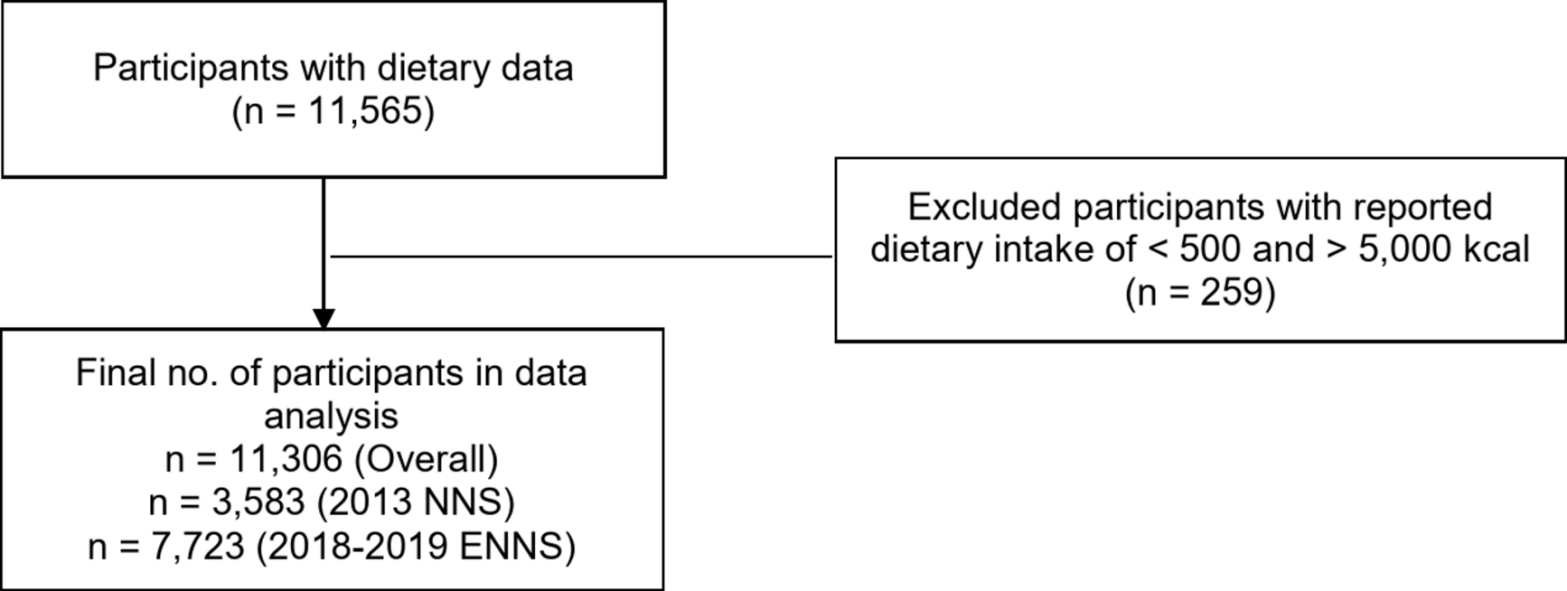
Participant inclusion and exclusion criteria flowchart

A total of 11,565 older adults aged 60 and above participated in the 2013 NNS and 2018–2019 ENNS with dietary data. Participants with an extreme dietary intake (< 500 or > 5,000 kcal) were excluded from the analysis [23–26]. A total of 11,306 participant data were included in the analysis, 3,583 for the 2013 survey and 7,723 for the 2018 survey (Fig. 1).

### Variables

#### Anthropometry

Weight was measured using a calibrated platform balance beam scale (Detecto™, 0.1 kg) and digital weighing scale (SECA™, 0.01 kg). Standing height was measured to the nearest 0.1 cm using Seca™ microtoise (2013) and stadiometer (2018). WHO cut-off point was used for body mass index (BMI) classification [27]. Trained anthropometry researchers performed all measurements.

#### Sociodemographic and lifestyle

Socioeconomic and demographic data were collected using an interview method which includes information on sex (male or female), age (60–69 years old or 70 years and above), educational attainment (elementary level, high school level, and at least college level), and residence (rural or urban) among others. Socioeconomic status (SES) was defined by means of wealth quintile (very low income, low income, middle, high income, and very high income) using proxy indicators including household assets, household characteristics, type of dwelling unit, housing materials etc. Lifestyle information on smoking (currently smoking or not), and alcohol consumption (currently consuming alcohol or not) were gathered using interview method. WHO Global Physical Activity Questionnaire was used to classify physically active or inactive respondents [11, 21]. Trained researchers conducted all the interviews.

#### Dietary intake

For both survey years, usual dietary intake at the individual level was obtained by 2-day non-consecutive food recall through a face-to-face interview by trained nutritionist-dietitians. All respondents were interviewed for the 1 st day 24-hour food recall while 50% were randomly selected for the 2nd day 24-hour recall [11, 21]. Tools such as food photographs, labels and models were used to aid in quantification of portion sizes. Reported food portion sizes were converted into food weights expressed in grams.

### Dietary pattern and statistical analysis

For each survey, dietary pattern analysis was done independently. Dietary patterns were generated using Principal Component Analysis (PCA) [18, 28]. Food items were classified into 17 food groups (See Additional File 1 Table S1) based on the existing local food guides (Daily Nutritional Guide Pyramid, 2012 Nutritional Guidelines for Filipinos) and the food groupings from the NNS. PCA was carried out using varimax rotation to reduce the variance, improve interpretability, determine dietary patterns, and the factor loadings for each food group. Dietary pattern components with an eigenvalue ≥ 1.0 were retained and factor loadings of *≥* 0.25 or *≤ −* 0.25 were used to describe the components based on previous studies [16, 18]. Factor loadings describe the correlation of the food groups in a principal component wherein a positive value entails a positive correlation while a negative value means an inverse relationship in each food group in the component. Lastly, the derived dietary patterns were critically checked based on local customs and published results from the NNS in the country [11, 16, 18, 21]. Prior to the derivation of the dietary patterns by PCA, the Overall Measure of Sampling Adequacy was evaluated using the Kaiser–Meyer–Olkin statistic (> 0.50) while Bartlett’s test of sphericity (*p* < 0.000) was performed to evaluate the suitability of the survey datasets (See Additional File 2 Table S2).

All analyses performed with *p*-value of < 0.05 were considered as statistically significant. STATA version 12.1 (Stata Statistical Software: Release 12. College Station, TX: StatCorp LP.) was used in the analyses. Data was checked for normality using visual QQ plots. Continuous variables were presented as means and standard deviations while categorical variables were presented as percentages. Adherence to the dietary pattern was treated as binary variable (low to medium adhering = 0, high adhering = 1). Using the factor scores from each dietary pattern, participants that belonged to the 66th percentile and below of the factor score were considered as low to medium adhering group while the high adhering group were those above the 66th percentile of the factor score. A similar principle of grouping an outcome variable into with 66th percentile as the reference cut-off point was done in a previous study [29]. Multivariate logistic binary regression was used to determine the association of adherence to the dietary pattern with sociodemographic factors and nutritional status. Independent variables included sex, age group, household wealth quintile, urban-rural residence, education level, BMI category, alcohol use, smoking status, and physical activity. These covariates were entered simultaneously in the multivariate logistic regression models syntax for each dietary pattern. To consider the complexity of the survey design of both national surveys, survey (*svy*) command in STATA was used to adjust for the sampling weights. Observed differences between the two surveys are presented descriptively without inference testing.

## Results

Table 1 shows that the overall mean age in years of older adult participants was similar for both the NNS period at 68.7 and 68.4 in 2013 NNS and 2018–2019 ENNS, respectively. More than half of the participants in both NNS years were female, 54.8% in 2013 NNS and 56.3% in 2018–2019 ENNS. The highest proportion (45.2% and 38.9%) of the participants were from the island of Luzon and the distribution in terms of residence was nearly equal for both survey years. In both surveys, most respondents reached at least an elementary level. A chronic energy deficient prevalence of 23.0% and 16.7% was observed from the 2013 to 2018–2019 NNS, while a prevalence of 25.2% and 30.9% in both overweight and obesity, respectively, was seen throughout the survey period. Nearly one-third of the population were current alcohol drinkers for both survey years while there were more current smokers in 2013 (22.3%) than in 2018–2019 (16.3%). In terms of physical activity, 54.0% of the respondents has low physical activity in 2013 while 48.9% in 2018–2019.

**Table 1:** Participant characteristics by NNS

	2013 NNS (*n* = 3583)			2018–2019 ENNS (*n* = 7723)		
		*LB*	*UB*		*LB*	*UB*
Age, years (mean, SD)	68.7	68.4	69.0	68.4	67.9	69.0
Sex (%)						
Male	45.2	43.8	46.7	43.7	42.4	45.0
Female	54.8	53.3	56.2	56.3	55.0	57.6
Age group (%)						
60–69	63.1	61.1	65.0	66.4	62.8	69.7
70 above	36.9	35.0	38.9	33.6	30.3	37.2
Wealth Index (%)						
Poorest	17.2	15.4	19.1	17.5	14.0	21.7
Poort	18.4	16.7	20.1	20.3	16.0	25.3
Middle	21.2	19.5	22.9	19.1	17.4	20.9
Rich	20.6	18.7	22.6	19.5	17.6	21.6
Richest	22.7	20.7	24.9	23.6	19.2	28.6
Group of islands (%)						
Luzon	45.2	42.1	48.4	38.9	17.1	66.4
Visayas	21.4	19.0	24.0	24.8	11.0	46.7
Mindanao	19.1	17.1	21.2	17.7	6.3	40.8
National Capital Region	14.3	11.6	17.5	18.6	7.1	40.7
Residence (%)						
Rural	50.8	46.7	54.8	55.6	39.4	70.6
Urban	49.3	45.2	53.3	44.4	29.4	60.6
Educational Attainment (%)						
Elementary	61.4	58.9	63.8	51.9	45.5	58.2
High School	22.9	21.3	24.7	26.4	22.7	30,5
College	15.7	13.9	17.8	21.7	18.6	25.1
BMI (%)						
Chronic Energy Deficient	23.0	21.6	24.6	16.7	14.9	18.7
Normal	51.8	49.8	53.7	52.4	49.9	55.0
Overweight and Obese	25.2	23.3	27.2	30.9	27.2	34.8
Alcohol consumption status (%)						
Current drinker	33.0	31.1	34.9	30.8	27.3	34.5
Smoking status (%)						
Current smoker	22.3	20.8	23.9	16.3	14.9	17.8
Physical Activity (%)						
Low	54.0	51.6	56.3	48.9	45.1	52.8
High	46.0	43.7	48.4	51.1	47.3	54.9

*LB* Lower Boundary, *UB* Upper Boundary

Variables with missing observations:

2013: wealth index (*n*=95), residence (*n*=65), education (n=15), alcohol drinking (*n*=193), smoking status (*n*=193), physical activity (*n*=231)

2018: wealth index (*n*=22), residence (*n*=22), education (*n*=19), alcohol drinking (*n*=154), smoking status (*n*=154), physical activity (*n*=85)

Three major dietary patterns were identified for the two NNS datasets using PCA (Table 2). The first dietary pattern was characterized by significant factor loadings on red meat and meat products, other cereals, sugar, poultry, eggs, and beverages and is labeled as meat-based pattern. The second dietary pattern, described as a traditional pattern, was composed of high positive loadings on rice and fish, and a negative loading on corn. The third pattern, labeled as vegetables, fruits and oil pattern was comprised of mainly vegetables, fruits, fats and oils, tubers, and corn. Based on the PCA results, the dairy and milk, condiments and miscellaneous, as well as beans and nuts food group were not part of the dominant dietary patterns of older adults.

**Table 2:** Factor loadings of the identified dietary patterns by NNS

Food groups	Dietary patterns and Factor loads^1^					
	2013 NNS			2018–2019 ENNS		
	Meat-based	Traditional	Vegetables, Fruits and Oil	Meat-based	Vegetables, Fruits and Oil	Traditional
Rice and rice-based products	−10	**63**	9	−3	9	**67**
Corn and corn-based products	−15	**−58**	15	−12	**26**	**−45**
Other cereals	**47**	−3	−6	**45**	−5	−11
Tubers	−4	−16	18	−4	**28**	−19
Sugars	**39**	2	12	**31**	−3	1
Beans, nuts, and seeds	12	2	0	15	17	3
Leafy vegetables	−20	−15	**50**	−14	**51**	2
Other non-leafy vegetables	0	−9	**48**	1	**51**	11
Fruits	8	8	**27**	9	**36**	−21
Fish and seafood	−24	**38**	7	−8	6	**37**
Red meat and meat products	**46**	−4	−6	**50**	−2	4
Poultry	**28**	6	7	**28**	4	5
Eggs	**25**	14	22	21	19	13
Dairy	23	−5	12	18	7	−21
Fat	11	17	**53**	15	**35**	20
Condiments and miscellaneous	−2	3	5	6	−2	−7
Beverages	24	−4	−7	**45**	−3	0
Proportion Variance^2^	8.9	8.5	7.6	9.1	7.8	7.8
Cumulative Variance ^2^	8.9	17.4	25.0	9.1	16.9	24.7

^1^Dietary patterns are labeled based on significant factor loadings (≥ 0.25) per principal component. Numbers are multiplied by 100 and in bold are the significant factor loadings

^2^Numbers are multiplied by 100 and rounded off to 1 decimal place

Table 3 reflects the association of nutritional status, sociodemographic and lifestyle factors for each derived dietary pattern by high- and low- to middle-adhering group. BMI and various sociodemographic and lifestyle factors were significantly associated with the three main dietary patterns. In both survey years, high adherence to the meat-based dietary pattern was significantly less likely among females and older adults. Specifically, females had lower odds of high adherence (OR = 0.70, 95% CI: 0.58–0.84 in 2013; OR = 0.88, 95% CI: 0.73–1.07 in 2018–2019), those aged 70 years and above (OR = 0.87, 95% CI: 0.72–1.04 in 2013; OR = 0.83, 95% CI: 0.70–0.97 in 2018–2019). Rural residence and lower socioeconomic status were also associated with lower odds of high adherence. For example, rural dwellers had ORs of 0.76 (95% CI: 0.62–0.94) and 0.73 (95% CI: 0.54–0.98) in 2013 and 2018–2019, respectively. Similarly, those in the poorest wealth quintile had substantially reduced odds (OR = 0.52, 95% CI: 0.97–0.72 in 2013; OR = 0.46, 95% CI: 0.37–0.58 in 2018–2019). In contrast, individuals with higher BMI were more likely to adhere to the meat-based pattern as well as those with higher educational attainment (OR = 1.77, 95% CI: 1.36–2.31 in 2013; OR = 2.03, 95% CI: 1.64–2.52 in 2018–2019).

**Table 3:** Association of sociodemographic and lifestyle factors and body mass index by dietary patterns using multivariate logistics regression for 2013 and 2018–2019 ENNS

	Meat-Based		Traditional		Vegetables, Fruits and Oil	
	2013 NNS	2018–2019 ENNS	2013 NNS	2018–2019 ENNS	2013 NNS	2018–2019 ENNS
Low Adhering Group (reference) vs. High Adhering Group						
Male (reference)						
Female	**0.70 (0.58–0.84)**	**0.88 (0.73–1.07)**	**1.45 (1.27–1.65)**	**1.52 (1.25–1.84)**	0.85 (0.73-1.00)	**0.40 (0.33–0.49)**
60–69 years (reference)						
70 years above	0.87 (0.72–1.04)	**0.83 (0.70–0.97)**	1.16 (0.98–1.36)	1.01 (0.88–1.16)	0.97 (0.81–1.17)	**0.67 (0.58–0.76)**
Urban (reference)						
Rural	**0.76 (0.62–0.94)**	**0.73 (0.54–0.98)**	**0.60 (0.50–0.73)**	0.95 (0.77–1.09)	**1.23 (1.01–1.49)**	1.28 (0.96–1.70)
Poorest	**0.52 (0.97 − 0.72)**	**0.46 (0.37–0.58)**	0.94 (0.72–1.24)	1.35 (0.93–1.96)	1.19 (0.90–1.56)	1.02 (0.82–1.27)
Poor	0.81 (0.61–1.09)	**0.64 (0.49–0.84)**	0.93 (0.72–1.19)	1.04 (0.86–1.25)	1.06 (0.82–1.38)	1.04 (0.85–1.26)
Middle (reference)						
Rich	**1.52 (1.15–2.01)**	1.20 (0.89–1.62)	1.14 (0.88–1.49)	1.17 (0.91–1.52)	1.28 (0.97–1.68)	0.82 (0.55–1.22)
Richest	**2.10 (1.60–2.77)**	**1.52 (1.08–2.15)**	**1.64 (1.24–2.16)**	1.37 (0.93–2.02**)**	**1.34 (1.01–1.77)**	0.92 (0.60–1.42)
Elementary (reference)						
High School	**1.62 (1.32–1.99)**	**1.55 (1.30–1.85)**	**1.32 (1.07–1.63)**	1.26 (0.96–1.66)	1.23 (0.98–1.53)	0.97 (0.81–1.17)
College	**1.77 (1.36–2.31)**	**2.03 (1.64–2.52)**	**1.75 (1.32–2.30)**	**1.68 (1.27–2.23)**	**1.89 (1.41–2.52)**	1.10 (0.82–1.46)
Below normal BMI	0.83 (0.67–1.04)	0.90 (0.76–1.08**)**	1.18 (0.98–1.42)	1.03 (0.84–1.27)	0.86 (0.70–1.06)	0.77 (0.54–1.08)
Normal BMI (reference)						
Above normal BMI	**1.29 (1.07–1.56)**	**1.19 (1.02–1.38)**	0.91 (0.73–1.12**)**	0.82 (0.66–1.01)	1.09 (0.90–1.34)	1.10 (0.87–1.39)
Non-Drinker (reference)						
Current Alcohol Drinker	1.00 (0.82–1.22)	**1.62 (1.34–1.98)**	1.17 (0.97–1.41)	1.00 (0.70–1.44)	1.16 (0.96–1.42)	1.02 (0.80–1.31)
Non-Smoker (reference)						
Current Smoker	1.04 (0.84–1.30)	1.18 (0.94–1.49)	0.87 (0.71–1.06)	**0.78 (0.62–0.98)**	0.93 (0.76–1.52)	1.06 (0.89–1.27)
Low Physical Activity Level (reference)						
High Physical Activity	0.98 (0.81–1.19)	**0.88 (0.77–0.99)**	1.06 (0.88–1.26)	1.04 (0.86–1.24)	**1.29 (1.09–1.53)**	1.17 (0.99–1.26)

The values presented are odds ratio (95% confidence intervals)

Values in bold are significantly different at a level of *p* < 0.05

For the traditional dietary pattern, high adherence was more likely among females, individuals with higher socioeconomic status, and those with higher educational attainment. Females had higher odds of adherence (OR = 1.45, 95% CI: 1.27–1.65 in 2013; OR = 1.52, 95% CI: 1.25–1.84 in 2018–2019), while those with college education had ORs of 1.75 (95% CI: 1.32–2.30) in 2013 and 1.68 (95% CI: 1.27–2.23) in 2018–2019. Adherence was also significantly associated with higher SES in the 2013 survey (e.g., richest quintile OR = 1.64, 95% CI: 1.24–2.16), though the trend was less consistent in 2018–2019. In contrast, current smokers was associated with lower adherence in 2018–2019 (OR = 0.78, 95% CI: 0.62–0.98), and rural residence was associated with lower OR only in the 2013 survey (OR = 0.60, 95% CI: 0.50–0.73). Other factors such as age, BMI, alcohol consumption, and physical activity showed no significant association with this pattern in either survey year.

For the vegetable, fruit, and oil pattern, the adherence was consistently higher among those with higher education and higher physical activity levels, but lower among females and older adults. For example, participants with college education had higher odds of adherence in 2013 (OR = 1.89, 95% CI: 1.41–2.52), and those with high physical activity also showed greater adherence (OR = 1.29, 95% CI: 1.09–1.53 in 2013). Conversely, females were significantly less likely to adhere to this pattern in 2018–2019 (OR = 0.40, 95% CI: 0.33–0.49). Adherence was also lower among those aged 70 and above in the 2018–2019 survey (OR = 0.67, 95% CI: 0.58–0.76). Rural residence and current smokers were positively associated with adherence only in the 2018–2019 survey, while SES showed mixed associations across survey years.

## Discussion

This study aimed to describe the dietary patterns and explore associated factors among Filipino older adults. Results of the PCA revealed three major dietary patterns that were similar for both the 2013 NNS and 2018–2019 ENNS. The first dietary pattern, described as meat-based, was composed of red meats, internal organs, poultry, eggs, snacks, condiments and beverages. The second dietary pattern was characterized by high loading on rice and rice-based products and fresh fish or seafood while negative loading on starchy roots and tubers and is labeled as the traditional pattern. The third pattern, labeled as a mixed diet, was comprised of roots and tubers, vegetables, fruits, fats and oils, fish/seafood and eggs. We found an association, to varying extent, between sociodemographic factors and nutritional status across all derived dietary patterns.

In this current study, our results agree with the works of Agdeppa et al. [18] and De Juras et al. [16] in which the dietary patterns derived among 20 years and above were mainly meat-based, traditional rice and fish, and a combination of vegetable, fruits and oil. The dietary patterns among older Filipino adults are like of the general Filipino adult population and remained relatively similar for both survey years. The similarity of the dietary patterns between the two surveys may be explained by the very distinct diet of Filipinos. Over the years, the typical Filipino diet has been rice, fish and vegetables with slight increase intake of protein-rich foods while low consumption of fruits, milk and dairy products, and beans and nuts [30]. Furthermore, our result seemed to be in contrary to the previous work that showed differences in dietary patterns among adults and older adults [31]. We speculate that the similarity in the derived dietary patterns of older Filipino adults with younger adults aged 20 years and above may be driven by the scenario that Filipino families would opt to provide care themselves for the older adults and have them stay in the same household and more likely older adults will consume the same food prepared for the entire household [32].

Noteworthily, for both 2013 NNS and 2018–2019 ENNS, we observed the minimal contribution of milk and dairy as well as beans and nuts food group in the dietary pattern of older Filipinos. The 2012 Nutritional Guidelines for Filipinos [33], *Pinggang Pinoy*^®^ [34] and Daily Nutrition Guide Pyramid recommends a varied and balance diet composed of grains or equivalent, lean meat, fish or protein alternatives (e.g., beans, nuts), calcium-rich foods, vegetables and fruits. Results from the surveys revealed that milk and dairy contributed only 5.6% and 1.2% to the overall intakes of older adults in 2013 and 2018, respectively. Meanwhile, beans and nuts constituted only 0.8% of the over diet in 2013 and 0.9% in 2018 [11, 21].

The Philippines is a low milk-drinking country and believed to have high prevalence of lactose intolerance [35, 36] such is the case in Indonesia were two-thirds of the Indonesian older adults are found to be lactose intolerant [37]. Furthermore, this may also be driven by financial constraints [38] since milk and dairy products are relatively expensive commodities in the country. Consumption of dairy products is found to be associated with a lesser risk of frailty [39] and sarcopenia [40] in later life.

Beans and nuts are considered as an alternative to animal protein for a lower cost. However, the low consumption among our population might be influenced by the common perception in the country that beans and nuts may trigger arthritis or other similar conditions common among the ageing population [41]. More so, the limited local dishes in the Philippines using beans and nuts as primary ingredient might also be a factor. In a review of Zheng et al. [42], replacing animal-based protein with plant-based protein found a strong inverse association on the onset of cardiovascular disease (CVD) and all-cause mortality. As such, it is worthwhile to revisit the dietary guidelines for older adults [43] in the country and consider in its development, implementation, and communication phases as part of the nutrition education for older adults to help promote and increase the consumption of milk, dairy products, beans and nuts as part of a healthy diet.

We found an association, in varying extent, among sociodemographic factors, nutritional status, and the derived dietary patterns. It is notable that sex is significantly associated with all dietary patterns for both surveys. Being a female has lower odds in adhering to a meat-based pattern and vegetable, and fruits, and oil pattern while higher odds in adhering to a traditional pattern of rice and fish. Females generally tend to make healthier food choices which include lesser meat and beverages intake as compared to male counterparts. Several studies have supported the notion that females are more cautious in food choices due to stronger belief in healthy eating and involvement in health and weight matters [44, 45]. A time trend study among Taiwanese adults showed that females have reduced risk for metabolic syndrome due to their adherence in relatively healthier dietary choices [46]. In addition, we observed that females have higher adherence to the traditional rice and fish pattern. Increased fruit and vegetable intake is commonly associated with healthier choices. It is interesting that our result showed that females have lesser odds in adhering to the vegetables, fruits and oil pattern for both surveys than the male counterpart which contrasts with the findings among Thai older women [47]. This can be plausibly attributed to the common local meal preparation. In general, a one-dish meal, such as *tinola*,* afritada*,* menudo*,* sinigang*, is a combination of meat (e.g. poultry, pork, beef) and vegetables and commonly prepared in households [48]. With older male Filipinos adhering more to a meat-based pattern, their reported diet might possibly reflect more vegetables consumed than the females. This is also evident in the 2013 NNS and 2018–2019 ENNS dietary report [11, 21].

In this study, age was associated with the meat-based pattern for both survey years. The 70 and above age group has lesser odds to adhere to a meat-based pattern than the 60–69 years group. The meat-based dietary pattern was composed of red meats, internal organs, poultry, eggs, snacks, condiments and beverages. These food items are commonly avoided due to their possible contribution to the onset of age-related non-communicable diseases such as CVD, diabetes, hypertension and cancer. Furthermore, age is a known major risk factor in the onset of diseases [49, 50]. As we age, bodily function alters which poses risk to one’s health and life [51, 52]. Our results were in line with previous studies [14, 53] documenting the relationship of increased age and healthier food choices. A study among outpatients in Italy found differences in dietary pattern between younger and older adults with the older group adhering to a healthier pattern of the Mediterranean diet [31]. Furthermore, in a focus group discussion conducted among Thai older adults [54], increase in age was found to be a determinant of healthy food choices and eating practices.

The strong relationship between educational attainment and wealth is acknowledged [55]. Interestingly, it was only in the 2013 NNS that we found an association of high SES and educational attainment with higher adherence to fruits, vegetables and oil pattern while a higher adherence was observed among the lower SES and educational attainment in the 2018–2019 ENNS. A plausible explanation in the observed shift in the association from 2013 NNS to ENNS was the increased efforts in the campaign and implementation on backyard gardening and food security programs in the country [56, 57]. In 2018, the Nutrition month theme in the Philippines set by the National Nutrition Council was “*Ugaliing magtanim*,* Sapat na nutrisyon aanihin*” which mainly focused on the role of agriculture and food security on proper nutrition [58]. Efforts on that year from both the national and local level might have benefited the households with lower SES and educational attainment to have access to vegetables and fruits explaining such result. Collectively, our results demonstrated the premise that higher income and educational attainment group tends to have access and have increased consumption of red meats [59, 60], and vegetables and fruits [61, 62], than those in the lower SES and education level. This calls for action in preventive intervention as early as young to mid-adulthood [63] to achieve healthy ageing. This should also be coupled with an enabling environment across all life stages including the older persons and their care givers to get sufficient nutrition and health information particularly on those with socio-economic disadvantages.

In recent years, the Philippines, considered an emerging market with economic growth potential, is moving towards the path of industrialization and urbanization [64]. Our study revealed that older persons residing in urban areas were found to adhere more to a meat-based pattern as compared to their rural counterparts. A study among Chinese older adults showed that those resided in the urban areas consumed more red meats [65]. Previous literature raised the concern on rapid urbanization and increased prevalence of NCDs among adults [66, 67]. It is imperative that nutrition and food environment are not overlooked amidst the effort of the country in keeping up with modernization and urbanization.

Regarding nutritional status and lifestyle, adherence to a meat-based pattern was higher among those with above-normal BMI and high physical activity levels. This is corroborated by results of other studies reporting that older adults consuming more red meats, processed meats, refined carbohydrates, and beverages was associated with high BMI [18, 45, 68]. Furthermore, published evidence supports our findings that older adults embracing a healthy lifestyle including increased physical activity has decreased adherence to the meat-based dietary pattern [62, 69].

To the best of our knowledge, this is the first study in the Philippines that delved into the dietary patterns of older adults aged 60 years and above using two nationally represented surveys and provided general foundation on the factors associated with each dietary pattern. The strength of this study is that we used two NNS data sets ensuring proper representation of our target population. Data collection procedures were conducted by trained researchers following standard procedures. Furthermore, our findings showed reproducibility as these are aligned with previous research done [16, 18]. In addition, PCA factors are treated as continuous variables in a regression model providing a useful advantage in further analysis [70]. However, to understand the result in a meaningful manner, limitations of this study should be acknowledged. Results between the two surveys were observed differences presented descriptively without inference testing. The dietary recall method used is prone to different measurement errors and heavily relies on the participant’s memory and estimation which can be a crucial factor among older adults. The data collection for the ENNS is intended to span from 2018 to 2021. However, this paper only worked on the data collected before the start of the COVID-19 pandemic. Moreso, the study design cannot draw any causation between the variables of interest and a longitudinal study is recommended to explore the association of dietary patterns with health and nutritional outcomes of older adults. Another point to raise is the explained variance determined by a posteriori technique such as PCA is relatively low (25.0% for 2013 NNS, 24.7% for 2018–2019 ENNS) due to variability of dietary data. However, our reported variance is in line with other local and international studies (21.7%, 20.9%, 24.2%, 24.9%) [16, 19, 71, 72]. Variance is also affected by the number of food groups used in the analysis. Despite the relatively low number of food groups used in this study, the groupings were aligned with the existing cultural food guides in the country and those used in the NNS. Ultimately, with the growing interest in dietary pattern studies, other statistical approaches such as latent class analysis and cluster analysis may be explored along with creation of nutrition and health models. While our multivariate analysis was able to adjust for confounding variables and covariates, the study’s exploratory nature should be emphasized. Future studies using multivariate models and longitudinal designs are warranted to validate and expand on these findings. Nonetheless, this study provides valuable insight and contributes to the limited body of evidence on aging nutrition in developing and emerging economies with relatively young populations, such as the Philippines.

## Conclusion

Our study revealed three major dietary patterns namely, meat-based pattern, traditional rice and fish pattern, and vegetables, fruits and oil pattern, among Filipino older adults from the 2013 NNS and 2018–2019 ENNS. The milk and dairy products, and beans and nuts food group are not present in any of the derived dietary patterns. The dietary patterns of older Filipinos were associated with sociodemographic characteristics and nutritional status. For both surveys, the odds of adherence to a meat-based pattern were significantly lower among females, adults aged 70 years and older, rural dwellers, individuals with low BMI, lower educational attainment, and lower socioeconomic status, as well as physically active older adults. Meanwhile, factors such as age, sex, BMI, and socioeconomic status were associated to varying extents with adherence to the traditional and vegetables, fruits and oil patterns. Collectively, our findings are valuable in crafting nutrition and health strategies, interventions, and policies as part of the overall social and health program of older adults in the country. As such, a combination of nutrition education and a enabling healthy food environment initiative that will encourage the consumption of vegetables and fruits, lean meats, fishes, legumes and nuts can be explored. Our study recognizes that a proper diet as part of a life stage intervention approach should be considered to properly inculcate the concept of proper nutrition and health starting adulthood. It is also recommended in countries such as the Philippines to put emphasis on the role of food and nutrition as part of their overall healthy aging framework of the country. Further local prospective study on the association of diet with health and nutritional outcome is warranted to strengthen the evidence base.

## Supplementary Information

Additional file 1. Table S1: Food groups.

Additional file 2. Table S2: Results of the Bartlett’s test of sphericity, Kaiser-Meyer-Olkin measure of sampling adequacy, scree plot of the components and eigenvalues and cumulative variance extracted after PCA.

## Data Availability

The data that support the findings of this study are available from the corresponding author upon reasonable request and with permission from the DOST-FNRI.

## Abbreviations

BMI: Body Mass Index
CVD: Cardiovascular disease
DOST: Department of Science and Technology
EA: Enumeration Areas
ENNS: Expanded National Nutrition Survey
FIERC: FNRI Institutional Ethics Review Committee
FNRI: Food and Nutrition Research Institute
NNS: National Nutrition Survey
PCA: Principal Component Analysis
PSA: Philippine Statistics Authority
SES: Socioeconomic status
