# Supplementary material for: Dietary patterns of Filipino older adults and associated factors: analysis of the 2013 National nutrition survey and 2018–2019 expanded National nutrition survey
Source: BMC Geriatr. 2025 Oct 23;25:800. doi: 10.1186/s12877-025-06426-2 (PMC12548240; doi:10.1186/s12877-025-06426-2)
Supplement: Supplementary file 1 — Additional file 1. Table S1: Food groups. [file 12877_2025_6426_MOESM1_ESM.docx]

**Additional file 1 Table S1: Food groups**

**Table S1. Food Groups**

| **Food Groups** | **Food Items Included** |
| --- | --- |
| Rice and rice-based products | - Rice, including processed products such as flours, noodles, popped rice, and native delicacies |
| Corn and corn-based products | - Corn, including processed products such as flours, noodles and popcorn |
| Other cereals | - Product from oats, whole wheat, including processed products such as flours, noodles etc. |
| Tubers | - Products from potatoes, sweet potatoes, yams, taro; excluding manufactured roots/tuber-based sweet/savory snacks |
| Sugars | - Sugars such as mono- and disaccharides, syrups, honey and others - Any other sweets such as jams, jellies, candies, bars, desserts |
| Beans, nuts, and seeds | - Products from fresh/dried beans, nuts and seeds excluding processed/manufactured products; including legumes consumed after basic household processing such as roasting, salting - Processed/manufactured legume products including canned, brined, drinks and other products |
| Leafy vegetables | - Includes all kinds of leafy vegetables - Processed/manufactured leafy vegetables including canned, concentrates, pastes, salted, pickled etc. |
| Other non-leafy vegetables | - Includes all kinds of non-leafy vegetables - Processed/manufactured non-leafy vegetables including canned, concentrates, pastes, salted, pickled etc. |
| Fruits | - Includes all kinds of fruits, fresh and processed, including dried, canned, brined, preserved etc., but excluding fruit juice/drinks, jams and jellies |
| Fish and seafood | - Fresh fish/seafood |
| Red meat and meat products | - Includes all kinds of red meat such as pork, beef, game, and their products - Offal from all kinds of red meat and poultry |
| Poultry | - Includes all kinds of poultry such as chicken, and game |
| Eggs | - Eggs from poultry or any other bird |
| Dairy | - Fresh liquid milk from cows or other mammals - Products derived from milk such as evaporated, powdered, fermented, cheese etc. |
| Fat | - Fats and oils of both plant and animal origin |
| Condiments and miscellaneous | - Herbs and flowers, fresh and dried, used as spices, and spices such as buds, seeds, root, fruit, powdered - Condiments, sauces and relishes such as soy sauce, vinegar - Powdered or instant soups/mixes, seasonings - Philippine FCT miscellaneous |
| Beverages | - Includes all kinds of beverages such as sugar-sweetened sodas, energy drinks, sport drinks, chocolate drinks, sweetened tea, fruit juices and other beverages such as coffee and tea |
