# Supplementary material for: Dietary patterns of Filipino older adults and associated factors: analysis of the 2013 National nutrition survey and 2018–2019 expanded National nutrition survey
Source: BMC Geriatr. 2025 Oct 23;25:800. doi: 10.1186/s12877-025-06426-2 (PMC12548240; doi:10.1186/s12877-025-06426-2)
Supplement: Supplementary file 2 — Additional file 2. Table S2: Results of the Bartlett’s test of sphericity, Kaiser-Meyer-Olkin measure of sampling adequacy, scree plot of the components and eigenvalues and cumulative variance extracted after PCA. [file 12877_2025_6426_MOESM2_ESM.docx]

**Additional file 2 Table S2: Results of the Bartlett’s test of sphericity, Kaiser-Meyer-Olkin measure of sampling adequacy, scree plot of the components and eigenvalues and cumulative variance extracted after PCA**

**Table S2.1 Results of the Bartlett’s test of sphericity and Kaiser-Meyer-Olkin measure of sampling adequacy from the 2013 NNS**

| Variables | Food groups, sex, age group, wealth quintile, place of residence, educational attainment, body mass index, alcohol drinking, smoking status |
| --- | --- |
| Determinant of the correlation matrix | **0.138** |
| Bartlett test of sphericity | |
| Chi-square | **6501.601** |
| Degrees of freedom | **300** |
| p-value | **0.000** |
| H0: variables are not intercorrelated | |
| Kaiser-Meyer-Olkin Measure of Sampling Adequacy | **0.651** |

**
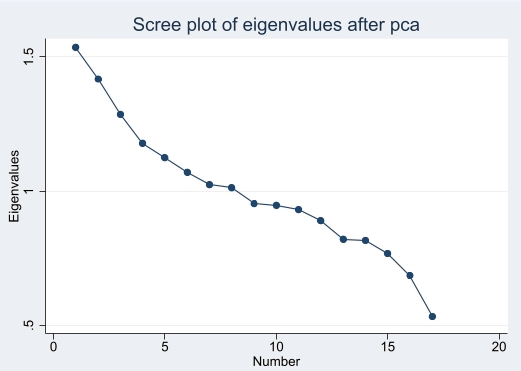
**

**Figure S2.1 2013 NNS Screeplot of the components extracted after principal component analysis**

**Table S2.2 Eigenvalues and cumulative variance of the components extracted after principal component analysis from the 2013 NNS**

| **Component** | **Eigenvalue** | **Cumulative** |
| --- | --- | --- |
| 1 | 1.53659 | 0.0904 |
| 2 | 1.41794 | 0.1738 |
| 3 | 1.28656 | 0.2495 |
| 4 | 1.17921 | 0.3188 |
| 5 | 1.12446 | 0.3850 |
| 6 | 1.07099 | 0.4480 |
| 7 | 1.02386 | 0.5082 |
| 8 | 1.01226 | 0.5678 |
| 9 | 0.953396 | 0.6238 |
| 10 | 0.948009 | 0.6796 |
| 11 | 0.930938 | 0.7344 |
| 12 | 0.889865 | 0.7867 |
| 13 | 0.821737 | 0.8350 |
| 14 | 0.816883 | 0.8831 |
| 15 | 0.767719 | 0.9283 |
| 16 | 0.68626 | 0.9686 |
| 17 | 0.533324 | 1.0000 |

**Table S2.3 Results of the Bartlett’s test of sphericity and Kaiser-Meyer-Olkin measure of sampling adequacy from the 2018-2019 ENNS**

| Variables | Food groups, sex, age group, wealth quintile, place of residence, educational attainment, body mass index, alcohol drinking, smoking status, physical activity |
| --- | --- |
| Determinant of the correlation matrix | **0.162** |
| Bartlett test of sphericity | |
| Chi-square | **13684.029** |
| Degrees of freedom | **325** |
| p-value | **0.000** |
| H0: variables are not intercorrelated | |
| Kaiser-Meyer-Olkin Measure of Sampling Adequacy | **0.657** |

**
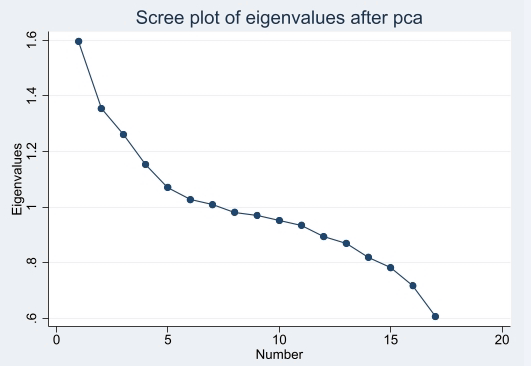
**

**Figure S2.2 2018-2019 ENNS Screeplot of the components extracted after principal component analysis**

**Table S2.4 Eigenvalues and cumulative variance of the components extracted after principal component analysis from the 2018-2019 ENNS**

| **Component** | **Eigenvalue** | **Cumulative** |
| --- | --- | --- |
| 1 | 1.5961 | 0.0939 |
| 2 | 1.35413 | 0.1735 |
| 3 | 1.26059 | 0.2477 |
| 4 | 1.15336 | 0.3155 |
| 5 | 1.07138 | 0.3786 |
| 6 | 1.02622 | 0.4389 |
| 7 | 1.00815 | 0.4982 |
| 8 | 0.982245 | 0.5560 |
| 9 | 0.970471 | 0.6131 |
| 10 | 0.951924 | 0.6691 |
| 11 | 0.93552 | 0.7241 |
| 12 | 0.893698 | 0.7767 |
| 13 | 0.870262 | 0.8279 |
| 14 | 0.820389 | 0.8761 |
| 15 | 0.781771 | 0.9221 |
| 16 | 0.717293 | 0.9643 |
| 17 | 0.606493 | 1.0000 |
